# Exploring new insights in coronary lesion assessment and treatment in patients with diabetes mellitus: the impact of optical coherence tomography

**DOI:** 10.1186/s12933-023-01844-1

**Published:** 2023-05-24

**Authors:** Tobias Michiel Hommels, Renicus Suffridus Hermanides, Enrico Fabris, Elvin Kedhi

**Affiliations:** 1 Isala Hospital, Zwolle, The Netherlands; 2 Cardiovascular Department, University of Trieste, Trieste, Italy; 3 Division of Cardiology and Structural Heart Diseases, Medical University of Silesia, Poniatowskiego 15, 40-055 Katowice, Poland; 4 Department of Cardiology, Hôpital Erasme, Université libre de Bruxelles, Route de Lennik 808, 1070 Brussels, Belgium

**Keywords:** Coronary artery disease, Diabetes mellitus, Vulnerable plaque, Thin-cap fibroatheroma, Optical coherence tomography, Bioresorbable scaffolds

## Abstract

In this review, we summarise new insights into diagnostic approaches and treatment strategies for coronary artery disease (CAD) in patients with diabetes mellitus (DM). Despite the improvements in therapy, the clinical management of DM patients remains challenging as they develop more extensive CAD at a younger age and consistently have worse clinical outcomes than non-DM patients. Current diagnostic modalities as well as revascularisation treatments mainly focus on ischemic lesions. However, the impact of plaque morphology and composition are emerging as strong predictors of adverse cardiac events even in the absence of identified ischemia. In particular, the presence of vulnerable plaques such as thin-cap fibroatheroma (TCFA) lesions has been identified as a very strong predictor of future adverse events. This emphasises the need for an approach combining both functional and morphological methods in the assessment of lesions. In particular, optical coherence tomography (OCT) has proven to be a valuable asset by truly identifying TCFAs. New treatment strategies should consist of individualised and advanced medical regimens and may evolve towards plaque sealing through percutaneous treatment.

## Introduction

The incidence and prevalence of diabetes mellitus (DM) are increasing and it has currently become the most common global metabolic disorder [1, 2]. DM is associated with an excess of mortality and morbidity [3]. DM is considered an independent risk factor for coronary artery disease (CAD) and people with DM are between two and four times more likely to develop CAD than those without DM [4–7]. Furthermore, DM is considered a strong cardiovascular risk factor given that DM patients without myocardial infarction (MI) have a 5-year cardiovascular mortality similar to that of non-DM patients with a history of MI [8, 9]. Furthermore, CAD is responsible for 80% of deaths and 75% of hospital admissions in DM patients [10].

DM patients are at increased risk for developing acute coronary syndrome (ACS). Consequently, this increased risk has resulted in DM having a prevalence of 25–40% among patients presenting with ACS [9, 11, 12]. In fact, DM appears to be the main independent predictor of death or MI in the setting of ACS [13]. These patients are also at higher risk for advanced atherosclerosis presenting as diffuse CAD with more complex angiographical patterns, characterised by multivessel plaques, extending to mid and distal branches, which in turn makes myocardial percutaneous revascularisation more challenging [13]. Therefore, DM patients presenting with ACS have a higher chance of poor clinical outcomes that persist after the implementation of best practice protocol mandated care [14–16].

Despite advances in percutaneous coronary intervention (PCI) with the utilisation of modern drug-eluting stents (DES), studies have continued to show a trend towards higher rates of adverse cardiovascular events in DM patients than non-DM patients presenting either with or without ACS [17–20]. The outcomes of the BARI and FREEDOM trials enforced the superiority of coronary artery bypass grafting (CABG) over PCI in DM patients with multivessel disease presenting with stable coronary disease or stabilised ACS [21–24]. Consequently, current evidence indicates that in DM patients with stable CAD suitable for both procedures with low predicted surgical mortality, CABG is superior to PCI in reducing the risk of major adverse cardiac events [25]. However, in DM patients with a low SYNTAX score (≤ 22), PCI with modern DES has achieved outcomes similar to CABG with regard to death, MI and stroke [25]. Therefore, PCI may represent an alternative to CABG for CAD with low complexity of coronary anatomy involvement. In addition, when these patients present with ongoing MI, the need for urgent revascularisation of the culprit lesions is easily achievable by means of PCI [26].

During the choice of the revascularisation strategy it is of crucial importance to acknowledge that patients with DM often present with CAD at a younger age than their non-DM counterparts. Therefore, maintaining these patients free of cardiac ischemia over a longer period of time becomes challenging. While CABG is indeed the treatment of choice for complex multivessel disease in this patient population, it is known that (venous) grafts also have a limited patency that hardly extends over a decade; therefore, PCI has an important role in delaying the time that these patients ultimately receive CABG [27]. In concurrence, the importance of medical treatment in this patient category is pre-eminent as was shown in the BARI 2D trial [28]. From this perspective, efforts to improve clinical outcomes in the early stage of CAD in DM patients have become paramount.

## Pathophysiology of coronary artery disease in diabetes mellitus

Multifactorial pathophysiological processes in patients with DM contribute to an increased risk for developing both CAD and ACS. Persistent hyperglycemia and insulin resistance induce metabolic disarrangements and oxidative stress through diverse molecular processes that stimulate the accelerated development, progression and instability of atherosclerotic plaques [29, 30]. These mechanisms activate multiple pro-inflammatory and pro-atherosclerotic functions in the endothelium, vascular smooth muscle cells and leucocytes. Furthermore, increased glucose concentrations generate protein advanced glycation end products that bestow changes in enzyme activity, cross-linking, proteolysis susceptibility, macromolecular recognition, endocytosis and immunogenicity.

**Fig. 1:**
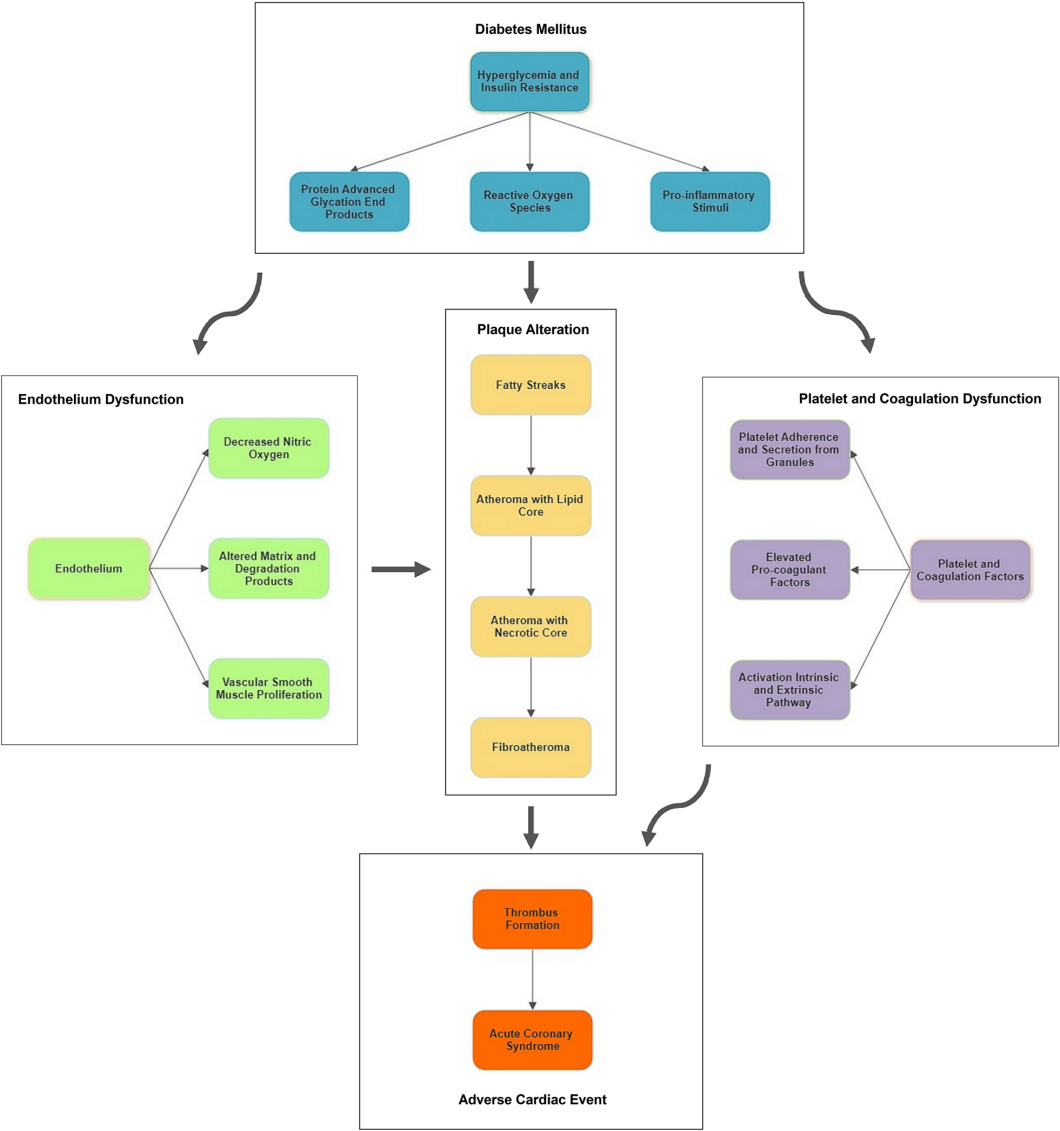
Pathophysiology of lesion instability in diabetes mellitus. Shown is the development of coronary artery disease in diabetes mellitus. Diabetes mellitus (blue) bestows endothelial dysfunction (green), plaque alteration (yellow) and platelet/coagulation disturbances (purple). The interplay between these factors leads to the development of fibroatheroma that is prone to rupture. In a pro-thrombotic environment this evolves into a substrate for thrombus formation and initiates an adverse cardiac event (red)

These complex disturbances are divided into three main functional categories: endothelial dysfunction, plaque alteration and platelet activation with coagulation disturbances. These processes are schematically illustrated in Fig. 1. In the endothelium, these disturbances lead to dysfunction with decreased nitric oxide production and increased production of reactive oxygen substrates. Vascular smooth muscle cells proliferate into the intima and develop both altered matrix components and degradation products that favour the composition of fibrosis. Plaque formation begins with the accumulation of lipids as fatty streaks in the vessel wall, which is accelerated due to the trapping of LDL particles in the altered environment. In more advanced stages, the accumulation of extracellular lipids leads to the formation of a lipid core, also known as an atheroma. Due to the impaired endothelial integrity and neovascularisation in the vasa vasorum, monocytes are able to penetrate and internalise these lipoproteins as cytokine-releasing macrophages, finally ending up as foam cells in the process. With an abundance of foam cells, a necrotic core is formed by repeated apoptosis, further contributing to the inflammatory state. In the final stages, these lesions develop a thin fibrous cap that turns into a fibroatheroma, which is more prone to rupture.

When the dysfunctional endothelium or the fibrous cap comes into contact with the bloodstream, it activates platelets that adhere to the vessel wall. The proposed mechanisms for this phenomenon are an interplay between diabetic-induced elevated concentrations of pro-coagulant factors and a reduction in endothelial antithrombotic properties in combination with the previously mentioned presence of reactive oxygen species and inflammatory stimuli. Activated platelets further release numerous mediators within their granules leading to an accelerated vicious cycle of plaque destabilisation. Finally, both intrinsic and extrinsic (by tissue factor-induced) coagulation pathways are activated, which produce a blood clot.

The presence of metallic devices may mechanically distort and constrain the stented segment of the coronary vessel, thus preventing the normalisation of vasomotion and autoregulation. In-stent restenosis may also be imposed by the presence of foreign material [31, 32]. This progressive late narrowing of the lumen is characterised by intimal hyperplasia and novel atherosclerotic plaque formation with prominent infiltration of lipid-laden macrophages, neovascularisation, fibrosis and the presence of proteinases at the struts while the surface containing the stent is covered by non-occlusive mural thrombi. The rupture of this newly formed plaque may contribute to late and very late stent thrombosis.

## Intravascular assessment of coronary artery disease

The currently most implemented catheterisation laboratory assessment of ischemic properties of coronary lesions is effectuated either by a visually directed quantitative coronary angiography or by hemodynamic measurements. Fractional flow reserve (FFR) is the most accurate technique to assess ischemia during hyperemia induced by adenosine injection; however, other rest pressure assessment techniques, such as the instantaneous wave-free ratio or resting full-cycle ratio are also widely adopted and do not require adenosine injection [33, 34]. For evaluating the ischemic burden of intermediate coronary stenosis, FFR measurements have led to more judicious utilisation of PCI and improved clinical outcomes [35, 36]. While FFR provides assessment of epicardial coronary stenosis severity and lesion-level ischemia, clinical events still occur even in patients with FFR > 0.80, with the possible explanation being abnormalities in the microvasculature [37]. Coronary flow reserve and the index of microcirculatory resistance may provide additional complementary information in such situations. Furthermore, evidence has suggested that in certain pro-thrombotic and pro-inflammatory conditions, such as in DM, deferring revascularisation on the basis of FFR guidance is associated with higher rates of adverse cardiac events than in patients without DM [38–41]. The reason for this phenomenon is that functional methods, such as FFR, lack the refinement to produce insights regarding morphological aspects of coronary atherosclerotic plaques and therefore are unable to detect differences in plaque inherent risks derived from plaque composition [42, 43].

### Vulnerable plaques

**Fig. 2:**
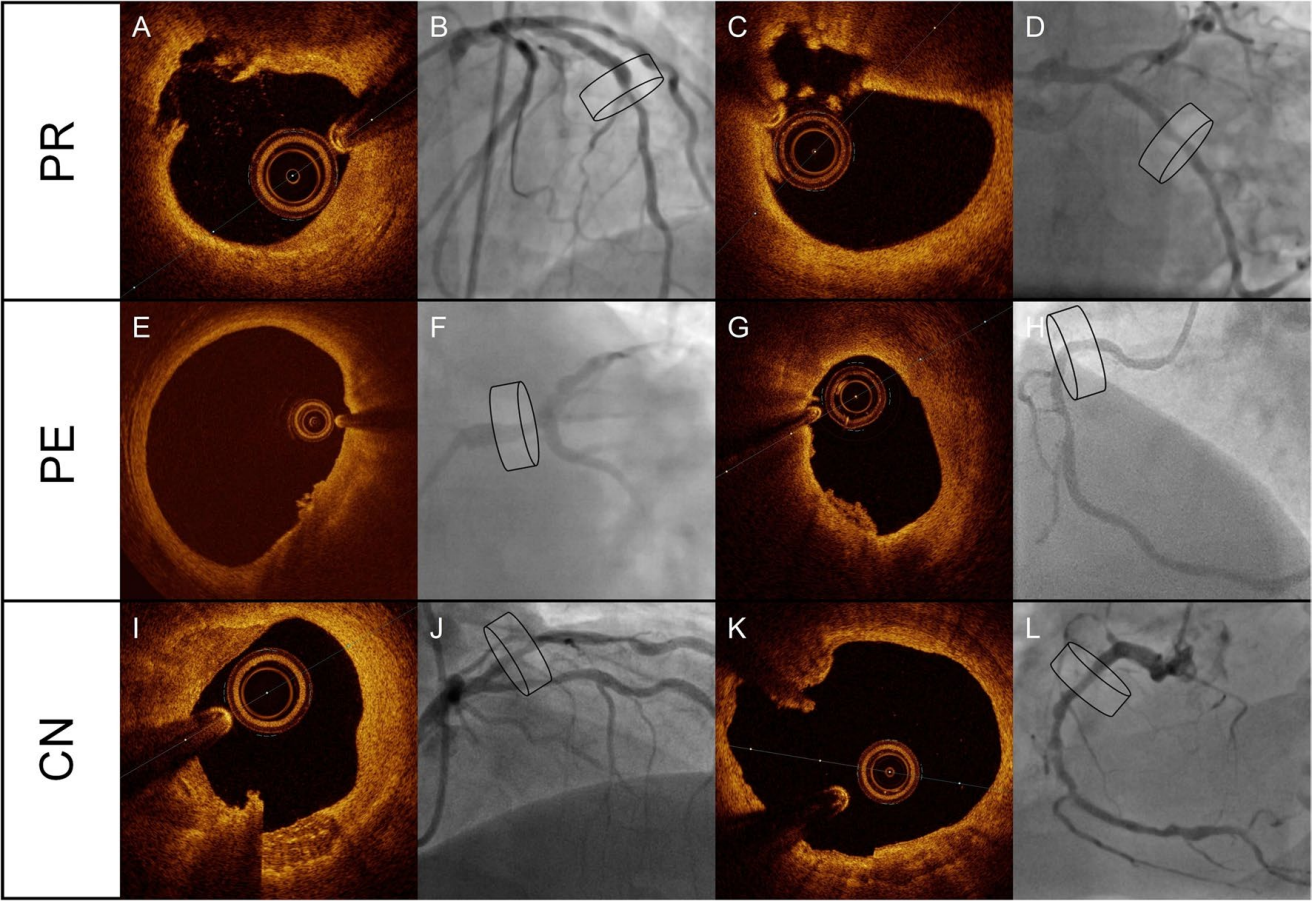
Major causes of acute coronary syndromes. The three major causes of thrombus formation are shown by optical coherence tomography-images coupled with corresponding coronary angiography, **A**,** B**–**C**,** D** plaque rupture (PR), **E**, **F**–**G**, **H** plaque erosion (PE) and **I**, **J**–**K**, **L** calcified nodules (CN). In the coronary lumen, an optical coherence tomography catheter and a guidewire providing shadow artefacts are present. Images AB visualise plaque rupture with the discontinuation of a fibrous cap and plaque cavity in the coronary artery. Images CD visualise plaque rupture with calcification, in which more plaque content has been washed away by the flush. Images EF display plaque erosion with the formation of a thrombus on an irregular luminal surface in the left main coronary artery. Images GH display plaque erosion without clear evidence of rupture. Images IJ present a calcified nodule that protrudes into the lumen of the coronary artery. Images LP present a protruding calcified nodule with luminal thrombus formation. The cylinders in the angiographic images indicate the location of the lesions

Historically, the concept of a vulnerable plaque was described several decades ago and opened a broad new era of research to enhance our knowledge of precursor determinants of adverse cardiac events [44]. In general, ACS is caused by the acute formation of a luminal thrombus on a designated atherosclerotic lesion with the exception of coronary spasm or spontaneous coronary dissection. Three distinct pathological causes of this phenomenon have been described: plaque rupture, plaque erosion and protruding calcification nodules [45]. These different entities are shown by intracoronary imaging with corresponding coronary angiography in Fig. 2. Plaque rupture is the most frequent cause, while plaque erosion and calcified nodules are more infrequent causes. Notably, these features do not necessarily originate from ischemic lesions. The term vulnerable plaques should be reserved for plaques that resemble precursor substrates for these major causes of acute thrombosis including pathological intimal thickening, plaque with spotty or nodular calcifications and the presence of thin-cap fibroatheroma (TCFA) (and to a lesser degree thick-cap fibroatheroma) [45]. The presence of a TCFA has mainly been associated with plaque rupture. Indeed, although in vivo histological analysis of the coronary vessel is an unfeasible option, the development of advanced intracoronary imaging makes characterisation possible with acceptable correlation with histological findings and clinical outcomes. For instance, the PROSPECT trial and ATHEROREMO-IVUS study determined that the presence of a TCFA, plaque burden > 70% and minimal lumen area (MLA) < 4 mm^2^ are independent predictors of adverse cardiac events [46, 47]. Nonetheless, adverse cardiac events rarely transpired in patients with non-fibroatheroma-carrying lesions, regardless of plaque burden and MLA values [46].

### The impact of thin-cap fibroatheromas

**Fig. 3:**
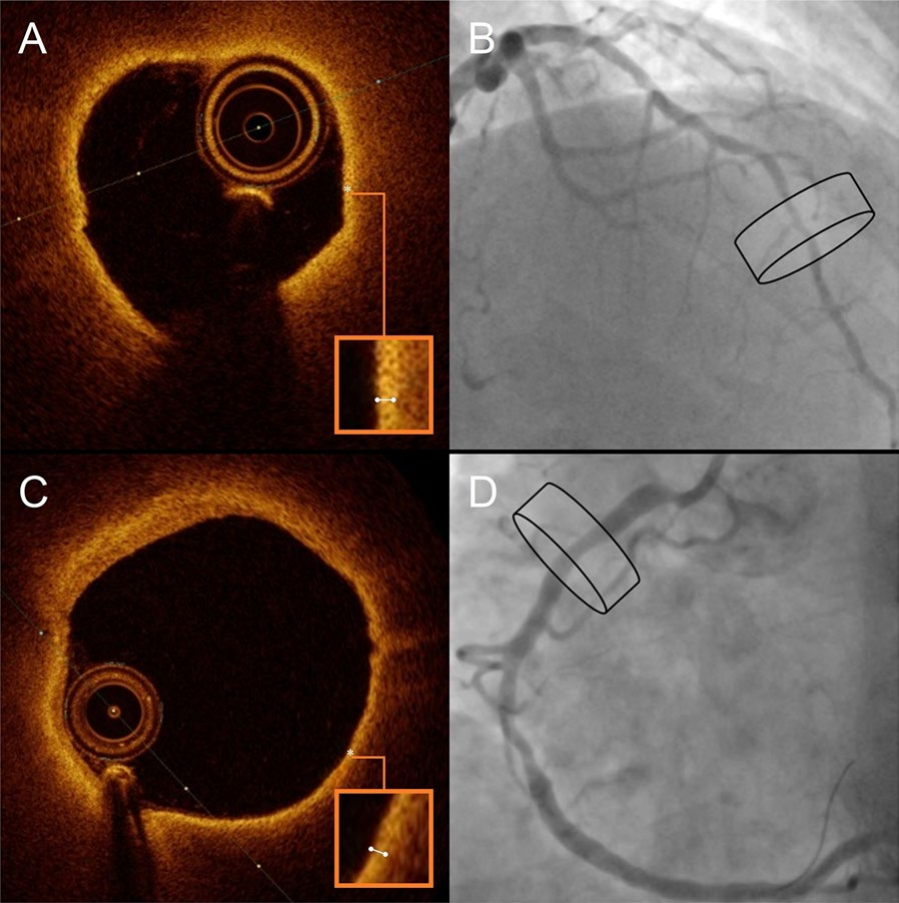
Thin-cap fibroatheroma in non-ischemic lesions. Optical coherence tomography images of thin-cap fibroatheromas (**A**, **C**) with the respective corresponding angiography (**B**, **D**) in patients with diabetes mellitus are shown. Thin-cap fibroatheromas are characterised by lipid-rich, non-calcified plaque with necrotic cores, in which the thinnest part of the fibrous cap measures ≤ 65 μm. A thin-cap fibroatheroma is the main precursor substrate for plaque rupture. In the optical coherence tomography images, lipid-rich plaques exhibit regions with poor signal and poorly defined borders, which are circularly present in both lesions. Part of the thinnest fibrous cap (shown with *) is magnified and measures ≤ 65 μm. The lesions were both of non-ischemic nature as determined by fractional flow reserve measurements. The cylinders in the angiographic images indicate the location of the lesions

A TCFA itself is defined as any coronary lesion with predominantly lipid-rich non-calcified plaque, a large necrotic core and foamy macrophage infiltration, for which the thinnest part of the atheroma cap measures ≤ 65 μm [45, 48]. TCFA plaque characteristics are shown in Fig. 3. TCFAs have been most frequently observed in patients who died because of acute plaque rupture (not plaque erosion) and usually occur in lesions with < 50% stenosis, demonstrating that TCFAs do not necessarily involve severe narrowing of coronary vessels [45]. Indeed, recent findings have shown that TCFA-carrying lesions are associated with rapid plaque progression as a consequence of silent plaque disruption and subsequent healing [49]. Risk factors for developing TCFA-carrying lesions include high total cholesterol, low high-density lipoprotein, a high total cholesterol/high-density lipoprotein cholesterol ratio and an increased pro-inflammatory state [45, 48].

Previous studies have shown that the prevalence of TCFAs in a population presenting with ACS is higher than that in patients with stable CAD, which may suggest that TCFA presence might be a strong predictor of culprit plaque rupture responsible for ACS [50]. These results are further supported by recent evidence demonstrating that the presence of TCFAs in non-culprit lesions was associated with developing future ACS and by the results of the CLIMA trial, in which an array of morphological factors for determining vulnerable plaques was evaluated [51, 52]. Furthermore, TCFA presence was found to be an independent predictor of future non-culprit lesion-related adverse cardiac events in patients with DM [41].

### Combining optical coherence tomography with fractional flow reserve

When evaluating the clinical consequences of CAD in DM patients, it is of great importance to uphold a distinction between ischemic symptoms and prognostic determinants. Under appropriate medical therapy, non-ischemic vulnerable lesions may still be relevant in terms of prognosis, while not all (symptomatic) ischemic lesions are deemed prognostically unfavourable, as was shown in a DM substudy of the ISCHEMIA trial [53]. This paradigm has demonstrated the need for a more elaborate assessment of coronary lesions by combined means of functional as well as morphological parameters in reference to these clinical outcomes.

Recently, the COMBINE OCT-FFR trial was conducted to address this approach of combined functional and visual assessment of coronary lesions [54]. This analysis addressed the important concept of whether optical coherence tomography (OCT) assessed TCFA presence predicts adverse cardiac events in the absence of ischemia in patients with DM and ≥ 1 de novo native coronary lesion with 40–80% visual diameter stenosis.

As predicted from this natural history study hypothesis, the results demonstrated that the presence of a TCFA in FFR-negative lesions was associated with a fivefold higher risk for adverse cardiac events. Second, TCFAs were present in only 25% of FFR-negative lesions but contributed to > 80% of adverse cardiac events. Finally, all new MI events originated from the TCFA lesions. Importantly, this increased risk of adverse events persisted during a long-term follow-up [55]. Furthermore, the study showed that the TCFA group had worse outcomes than the FFR-positive fully revascularised groups, although the study was not powered for assessing this particular endpoint. Interestingly, TCFA-carrying lesions with lower FFR values and smaller MLA might be at greater risk of rupture [56]. The main lesson learned from this study is that the presence of vulnerable plaque in angiographically intermediate or severe lesions is the main driver of dangerous future events even in the absence of ischemia. These findings are in line with those of another study that showed that while there is a correlation between FFR severity and plaque vulnerability, the majority of the event rates during follow-up are mainly driven by medically treated FFR-negative lesions with high vulnerability features [57]. These findings together provide new insights into the clinical impact of high-risk lesions and might lead to a substantial shift in the treatment of CAD.

### Intracoronary imaging

**Fig. 4:**
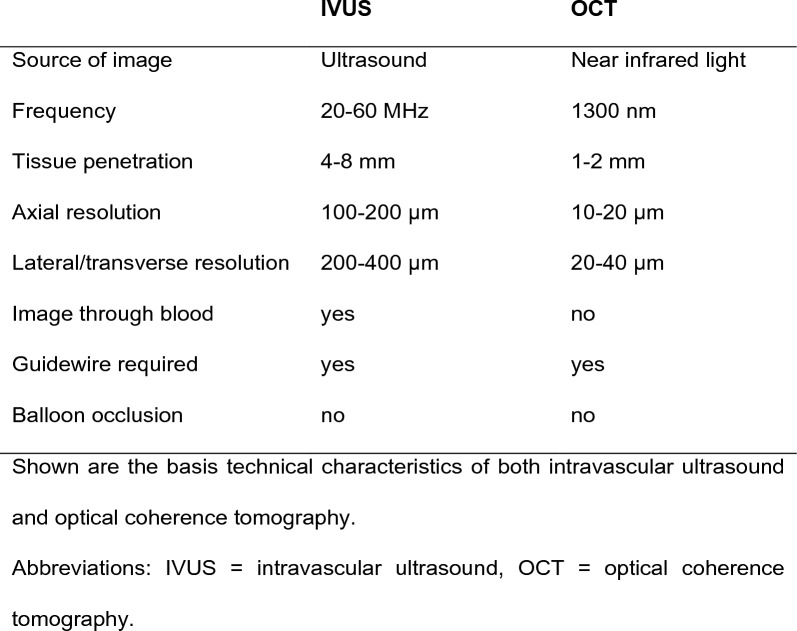
Characteristics of intravascular ultrasound and optical coherence tomography

**Fig. 5:**
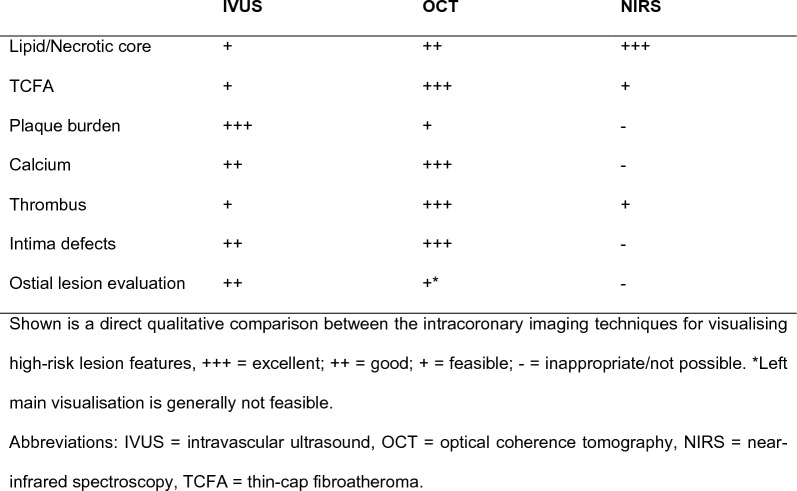
Capability of intracoronary imaging to detect high-risk plaque features

Both intravascular ultrasound (IVUS) and OCT have been implemented for visualising vulnerable plaques [58]. In addition, near-infrared spectroscopy (NIRS) could be utilised as an imaging technique in conjunction with IVUS as well as OCT. The intracoronary imaging modalities are described in Fig. 4 and their performance in detecting vulnerable plaque characteristics is presented in Fig. 5.

IVUS provides two-dimensional cross-sections that enable the visualisation of the lumen and stent struts as well as the dimensions of the coronary vessel wall. Despite its usefulness, IVUS remains limited in the assessment of certain plaque characteristics, for example lipid content, due to the inherent properties of sound waves, leading to a relatively low spatial resolution. On the other hand, NIRS imaging offers outstanding ability to detect a lipid-rich core in the plaque. This technology is based on the ability of tissue to absorb and scatter light at different intensities and wavelengths. However, NIRS alone also has limitations as it is unable to produce information about the lumen and plaque depth. The combination of IVUS and NIRS provides a hybrid imaging modality that is capable of detecting high-risk non-obstructive lesions with high lipid content and large plaque burden as was shown in the PROSPECT II trial [59]. However, even this combined imaging modality lacks the resolution to detect true TCFAs.

The OCT technique uses a low-coherence infrared light source that is directed at the vessel wall and produces tissue imaging with backscattered light. It does require the clearance of blood by contrast injection, as red blood cells are able to attenuate light. OCT has a higher spatial resolution than IVUS but less depth penetration. Therefore, OCT is currently the most accurate method for visualising and measuring the thickness of the fibrotic cap and thus detecting true TCFAs [60]. The relevance of this feature is paramount, as was also demonstrated in a substudy of the COMBINE OCT-FFR trial in which lipid-rich plaques were common but less likely to be associated with adverse cardiac events in the absence of TCFAs [61]. In this sense, OCT may substantially narrow the number of patients who might benefit from a more aggressive novel treatment. Importantly, methods other than OCT may overestimate the prevalence of TCFAs and thus vulnerable plaques. The utilisation of OCT for assessment of plaque burden is restricted given its limited tissue penetration into the layers of the coronary vessel, although implementation may still be feasible when interpreted by experienced operators or when software algorithms are applied. Furthermore, while OCT certainly offers the possibility to visualise lipids and the necrotic core, this performance deteriorates in the presence of large amounts of calcification and is associated with inter-observer disagreement. Therefore, a complementary combination of OCT for the analysis of plaque structure with NIRS for the analysis of plaque composition may prove to be beneficial [62].

## Medical treatment strategies

Medical therapy is the treatment of choice for stable CAD in DM patients. The BARI 2D trial, and more recently, the ISCHEMIA trial showed the non-inferiority of a medical approach compared to percutaneous treatment [28, 53]. Notwithstanding, study outcomes in DM patients are consistently characterised by a high residual cardiovascular risk in secondary prevention despite improved treatment combinations. However, the interpretation of these results remains challenging, as changes in therapy goals for glycemic control, directed by the outcomes of the ACCORD, VADT and ADVANCE trials, and changes to target LDL-cholesterol levels in this same time interval, may have influenced these outcomes to a certain degree [63–65]. Second, the effects of newer glucose-lowering drugs are not accounted for in most trials. Third, randomised clinical trials aimed at optimising antiplatelet treatment exclusively in DM patients with CAD are scarce. Thus, these patients are mainly treated with regimens validated in studies including only variable proportions of DM patients. Finally, type 1 DM remains widely less explored than type 2 DM. In general, medical treatment is divided into lipid-lowering, antidiabetic and antiplatelet agents.

### Lipid-lowering therapy

Treatment with statin therapy has been widely proven to improve clinical outcomes, especially in the setting of secondary prevention. Although the direct effects of statin therapy on reducing coronary stenosis diameter are limited, the beneficial effects include creating plaque stability. In addition to their lipid-lowering properties, statins induce pleiotropic effects by enhancing endothelial function and decreasing inflammation. On a coronary level, data from longitudinal imaging studies suggest that atherosclerotic plaque morphology may change over time, as it is able to both gain and lose features of vulnerability [66–69]. It has been demonstrated that up to 75% of vulnerable plaques might evolve towards a more stable phenotype under treatment with high-intensity statin therapy [70]. Recently, the HUYGENS and PACMAN-AMI studies further extended these findings by demonstrating an additional beneficial effect of proprotein convertase subtilisin/kexin type 9 (PCSK9) inhibitors on plaque composition in non-culprit vessels in patients with prior MI [71, 72]. These studies demonstrated the potential mechanism of very low LDL cholesterol levels on both plaque regression and clinical outcomes. Furthermore, these trials emphasise the role of OCT as this may be the new method for monitoring plaque progression and/or regression. Henceforth, OCT assessment could identify vulnerable plaques that may benefit from a more tailored medical treatment that comprises newer and more potent lipid-lowering drugs and/or newer antidiabetic drugs with stronger cardiovascular effects. Therefore, larger randomised controlled trials are necessary to confirm these findings.

### Antidiabetic therapy

The pursuit of the optimal antidiabetic therapy is extensive. In addition to conventional antidiabetic agents, newer glucose-lowering drugs have also been shown to reduce the incidence of major cardiovascular events as was shown in the LEADER, SUSTAIN-6 and EMPA-REG trials [73–75]. Glucagon-like peptide-1 (GLP1) agonists and sodium glucose cotransporter-2 (SGLT2) inhibitors have become the contemporary cornerstones for antidiabetic therapy in DM patients with CAD.

There are multiple substrates of GLP1 agonists, as they are responsible for glucose-dependent secretion of insulin, the inhibition of glucagon secretion and delayed stomach transit, all effects that favour cardiovascular protection. Indeed, these beneficial cardiovascular effects are unlikely to be driven only by a modest glycemic difference but rather by lowering blood pressure, body weight loss and by exerting favourable effects on lipid profiles [76, 77].

SGLT2 inhibitors are active by inducing reversible inhibition of the SGLT2 transporter with the result of glucose secretion and osmotic diuresis that in turn reduces preload and afterload with particularly favourable effects on heart failure. The beneficial cardiovascular protective effects might be related to the preservation of kidney function, increased uric acid excretion, reduction of epicardial adipose tissue, improved endothelium function and more efficient mitochondrial activity [76, 77]. Indeed, more evidence is emerging that SGLT2 inhibitors may have ameliorative effects on glucose homeostasis and advantageous pleiotropic glucose-independent effects on coronary plaque development. A recent study showed that DM patients with multivessel non-obstructive CAD treated with SGLT2 inhibitors had significantly higher values of OCT-detected fibrous cap thickness, fewer lipid deposits (measured as the lipid arc) and less macrophage infiltration than DM patients not treated with SGLT2 inhibitors [78]. In addition, a lower incidence of major adverse cardiovascular events was observed in this patient group [78]. Furthermore, in other studies with DM patients treated by PCI after MI, SGLT2 inhibitors were associated with a reduction in both safety outcomes and in-stent restenosis-related events [79, 80]. Consequently, regimens with SGLT2 inhibitors could result in the stabilisation of coronary plaques by reducing inflammatory stimuli and modulating fibrous cap thickness, leading to a reduction in adverse cardiac events.

### Antiplatelet therapy

The benefit of more potent antiplatelet therapies has been shown consistently in various clinical settings [81]. To a greater extent, a subgroup analysis of the PEGASUS-TIMI 54 study showed that in DM patients with prior MI, prolonged dual antiplatelet therapy (DAPT) regimens had additional benefits on cardiovascular endpoints at the expanse of a higher occurrence of bleeding events [82]. The THEMIS trials showed similar results in lower-risk DM patients with stable CAD without prior MI (with or without a previous PCI procedure) [83, 84]. Careful evaluation of thrombotic risk versus bleeding risk is a necessity as tools for guiding these decisions have been developed, for example the DAPT score which is also validated for use for DM patients [85].

Identifying patients with higher clinical risk is also possible by determining the duration and control of DM. Advanced DM that requires insulin prescription has been previously identified as a risk factor for the development of major adverse cardiovascular events in both FFR deferred lesions as well as those treated with PCI [29, 42, 53, 86]. Additionally, HbA1c levels may act as a surrogate for measuring current DM control until a better alternative becomes available. As the duration and current severity of DM may be related to cardiovascular complications, it appears reasonable to make efforts to strive for better glycemic control and to consider this especially when opting for a PCI treatment strategy. Following the ESC guidelines, HbA1c targets should be individualised according to age and co-morbidities, although levels below < 7% should be pursued [25]. Furthermore, there is observational evidence that HbA1c levels > 8% before PCI are associated with a higher incidence of adverse cardiovascular events, in particular MI and target vessel revascularisation [87]. Treatment with extensive DAPT regimens after previous PCI yielded consistent beneficial results, irrespective of DM duration and HbA1c levels [88].

## Interventional treatment strategies

Percutaneous coronary treatment is still the most utilised strategy in DM patients presenting with MI as well as in those with stable CAD. Newer-generation DES are associated with better safety and efficacy outcomes than bare metal stents or first-generation DES [89–91]. Furthermore, a large pooled analysis has shown that clinical outcomes after PCI in DM patients are highly dependent on lesion complexity at baseline [20]. Simple lesions are associated with efficacy outcomes as similar to those of non-DM patients, while DM patients with complex lesions have significantly higher adverse cardiac rates than non-DM patients. These data suggest that PCI may have favourable outcomes in a well-selected group of patients with DM, provided the extent of disease is less complex and thus is consistent with the results from the SYNTAX trial [92]. The EXCEL trial showed the safety of this strategy in an all-comer population even in the presence of left main coronary stenosis [93]. Therefore, PCI is currently still performed in DM patients, particularly in single vessel disease as well as in multivessel disease with a low SYNTAX score (≤ 22) directed by ischemia-guided revascularisation by FFR measurements [25].

### Focal percutaneous treatment of non-obstructive high-risk lesions

The ISCHEMIA trial showed that performing ischemia-guided revascularisation reduces angina frequency and improves quality of life but is insufficient to reduce prognostic endpoints such as cardiovascular mortality and MI when compared to an approach with optimal medical treatment [53]. It must be mentioned that ischemia-guided PCI, as was the case in the ISCHEMIA trial, exclusively targets ischemic lesions which represent approximately only one-third of all the lesions assessed [57]. Interestingly, the large majority of vulnerable plaques are also left on medical treatment by this approach. As shown recently in the COMBINE OCT-FFR trial, these vulnerable plaques are associated with a high rate of adverse events despite the absence of ischemia [54].

Given the results from the COMBINE OCT-FFR trial and those of other previously mentioned studies, it is plausible that PCI treatment could be reserved for a small subgroup of lesions with an intermediate to severe degree of diameter stenosis and vulnerable features even in the absence of ischemia [51, 52]. While treating such non-obstructive lesions percutaneously is quite controversial and currently not recommended in international guidelines, the safety and efficacy of plaque sealing by PCI may be considered given the low adverse event rates after the implantation of modern metallic DES in all-comer populations. Additionally, it may lead to a further drastic reduction in post-PCI event rates following wider implementation of imaging-guided precise stenting techniques. Indeed, OCT provides valuable guidance for PCI, as was shown in the ILUMIEN III trial with higher stent expansion and procedural success than with standalone angiographic guidance and fewer edge dissections than with IVUS-guided treatment, although its superiority for clinical endpoints has yet to be confirmed in the ILUMIEN IV trial [94]. From this perspective, a combined approach of FFR and OCT with or without NIRS may gain increased usage to guide revascularisation therapy in the future, especially in DM patients. Two ongoing large randomised studies, the COMBINE-INTERVENE (NCT05333068) and INTERCLIMA (NCT05027984) trials, are already testing the hypothesis that OCT-detected vulnerable plaque guided revascularisation, either alone or in combination with FFR, is superior to revascularisation by FFR guidance alone.

### Biodegradable drug-eluting stents

Despite the major advantages of novel DES with good general performance, ongoing restenosis and thrombotic events remain problematic with metallic DES in these generally younger DM patients. Biodegradable polymer DES were introduced to counteract this mechanism by developing a polymer that is prone to dissolving with the advantage of only leaving the bare metal stent. Nevertheless, new-generation durable polymer DES have proven to be very thrombo-resistent and outperformed these biodegradable polymer DES, mainly in the setting of DM and ACS [95, 96]. In response however, a novel Abluminus stent, a biodegradable drug-eluting device mounted on a drug-eluting balloon that is designed to deliver sirolimus, has shown preliminary promise in the treatment of coronary lesions in DM patients and is being further investigated in the ABILITY Diabetes Global trial [97].

### Bioresorbable scaffolds

A major downside of metallic DES is that repeated percutaneous interventions at target lesions lead to a critical loss in vessel diameter, thus shortening the time span where CAD can still be managed with PCI. Confronted with these conditions, bioresorbable scaffolds (BRS) have been developed to overcome these shortcomings through completely resorption within three years, with the additional hypothesis that vessel restoration and late remodelling after (non-layering) implantation might be associated with more beneficial long-term outcomes than metallic DES.

The multicentre ABSORB DM Benelux trial evaluated the utilisation of BRS in an exclusively all-comer DM population [98]. If implantation was performed by experienced operators, the short- and midterm outcomes were acceptable, even when compared to the excellent performance of third-generation DES in the TWENTE and DUTCH PEERS trials [99]. However, the first long-term results from the ABSORB trials and AIDA trial were unfavourable for BRS in comparison to DES in which greater late lumen loss, target lesion failure and an increased risk of very late scaffold thrombosis were reported [100–102]. Importantly, the main reasons for this phenomenon included not only the mechanical properties of the scaffold but also the suboptimal implantation methods along with inadequate drug therapy duration. Therefore, better results were obtained with optimised implantation methods in addition to more committed intracoronary imaging usage and prolonged DAPT regimens [103–107]. Such prolonged DAPT prescriptions, to overlap the duration of scaffold resorption, are acceptable considering the low bleeding risk of these young patients and do not differ from contemporary recommendations for treatment with metallic DES. Last, the late effects of BRS after their complete resorption have been marginally investigated, although a landmark analysis showed no additional risk after complete scaffold resorption, providing a noteworthy advantage in the treatment of chronic CAD [108]. Therefore, although BRS are currently withdrawn from daily practice, the concept remains attractive as improved scaffolds with thinner struts and more bioprotective properties are being developed.

Considering the young DM patient group and the dynamic plaque formation of vulnerable (ruptured) thin-capped lipid-rich soft plaques such as TCFAs as well as the downsides of permanent foreign material in the vessel wall caused by metallic DES, more optimised BRS with safer profiles could yet be particularly appealing in these DM patients. A similar method was already harnessed in the PROSPECT ABSORB trial, in which non-obstructive lesions with high plaque burden underwent revascularisation through the implementation of BRS with favourable long-term angiographic results when compared to standalone medical therapy [109]. The results of this trial warrant further dedicated research to determine the potential benefit of such a strategy. Hypothetically, the utilisation of BRS for the treatment of non-ischemic TCFA lesions in younger DM patients is a captivating possibility, as it may prove to provide the best of both worlds by preventing potential adverse cardiac events with only the temporary presence of intracoronary foreign material while giving time for (advanced) medical treatment to further enhance plaque stability.

## Conclusions

In modern interventional cardiology, CAD in patients with DM remains challenging as these patients develop more extensive vessel disease at a younger age and have consistently worse clinical outcomes for all current treatment strategies. As most validated methods are well established to determine ischemic lesions, they are unsuited to distinguish non-ischemic high-risk morphological plaque features such as TCFAs. Henceforth, this paradigm emphasises the need for a combined approach of both functional and morphological methods in the assessment of CAD in this specific patient category. In particular, OCT has proven to be a valuable asset. The treatment of these non-ischemic high-risk lesions should include individualised medical treatment strategies and may evolve towards plaque sealing through PCI, in which hypothetically the implementation of improved BRS might prove to be particularly beneficial.

## Data Availability

Not applicable.

## Abbreviations

ACS: Acute coronary syndrome
BRS: Bioresorbable scaffolds
CABG: Coronary artery bypass grafting
CAD: Coronary artery disease
DAPT: Dual antiplatelet therapy
DES: Drug-eluting stents
DM: Diabetes mellitus
FFR: Fractional flow reserve
GLP1: Glucagon-like peptide-1
IVUS: Intravascular ultrasound
MLA: Minimal lumen area
MI: Myocardial infarction
NIRS: Near-infrared spectroscopy
OCT: Optical coherence tomography
PCI: Percutaneous coronary intervention
PCSK9: Proprotein convertase subtilisin/kexin type 9
SGLT2: Sodium glucose cotransporter-2
TCFA: Thin-cap fibroatheroma
